# Nanoscale Materials for Water Purification and Catalysis

**DOI:** 10.3390/nano15131019

**Published:** 2025-07-01

**Authors:** Michael Arkas, Ioannis Anastopoulos, Dimitrios A. Giannakoudakis, Ioannis Pashalidis

**Affiliations:** 1 Demokritos National Centre for Scientific Research, Institute of Nanoscience and Nanotechnology, 15341 Athens, Greece; 2 Department of Agriculture, University of Ioannina, UoI Kostakii Campus, 47040 Arta, Greece; ianastopoulos@uoi.gr; 3 Institute of Chemical Sciences, Faculty of Chemistry, Maria Curie-Sklodowska University, 20031 Lublin, Poland; dagchem@gmail.com; 4 Environmental & Radioanalytical Chemistry Lab, Department of Chemistry, University of Cyprus, Nicosia 1678, Cyprus; pspasch@ucy.ac.cy

## 1. Introduction

It is generally accepted that the nanoscale realm frontiers are situated below 100 nm at least in one dimension. In this domain, the conventional rules of chemistry and physics do not apply as in bulkier particles, and thus, untypical size-dependent properties arise. For instance, increased thermal and physical stability, an extremely large specific surface area compared to volume, accompanied by an equally dense pore network that leads to high reactivity. Thanks to these exceptional features, nanomaterials attracted the accelerated interest of a substantial number of scientists, which resulted in the diversity of applications: as sensors, controlled delivery agents, optoelectronic devices, energy storage systems, and most importantly, adsorbents of aqueous pollutants. A plethora of contaminants extending from inorganic heavy metals, halides, oxides, and radionuclides to organic petroleum byproducts, drugs, polychlorinated biphenyls (PCBs), dyes, surfactants, perfluorinated compounds, pesticides, and microbial bacteria, fungi, viruses, and parasites may be removed by practicing nanotechnology. As anticipated, a differentiation of methods and classes of nanomaterials has been developed as a tailor-made solution to each pollution type. The most common processes include adsorption, (photo)catalytic decomposition, nanofiltration, and decontamination from microorganisms. In the present collection, the two former procedures are discussed, as implemented by an exceptional variety of novel materials: magnetic nanoparticles [1], nanohybrids bearing dendritic polymers [2,3], graphene and carbon nanotube nanocatalysts [4], nano-sized ceramics [5], inorganic [6] and nanocomposite photocatalysts functionalized by surfactants [7], aerogels [8], biochar [9,10], activated carbon doped by metals [11], and metal oxides [12]. All these suggestions represent sustainable and pioneering methods to enhance adsorption capacity and (photo)catalytic degradation efficiency, promoting, in parallel, the selectivity towards specific aqueous contaminants.

## 2. An Overview of the Published Articles

“Differentiating Nanomaghemite and Nanomagnetite and Discussing Their Importance in Arsenic and Lead Removal from Contaminated Effluents: A Critical Review” focuses on these iron oxides’ different magnetic properties and catalytic profiles. The need to fine-tune the applied magnetic field and surface functionalization [13] to achieve optimal performance and reusability after a certain number of adsorption/degradation–regeneration cycles is also highlighted [14].

Catalytic Neutralization of Water Pollutants Mediated by Dendritic Polymers provides an overview of the catalysis methods that utilize the contributions of dendrimers, hyperbranched polymers, and dendrons. Emphasis has been placed on hybrid organic–inorganic composites containing ceramic substrates [15] and metal nanoparticles [16].

“Carbon-Based Nanocatalysts (CnCs) for Biomass Valorization and Hazardous Organics Remediation” emphasizes the (sono)photocatalytic decomposition of pollutants [17] and the production from biomass, via analogous methods, of carbonaceous catalytic nanoparticles that address the problem of toxic substance removal [18]. An application-specific description of the desired characteristics, i.e., size, porosity, specific surface, and external group functionalities, is offered as well.

“Uranium Removal from Aqueous Solutions by Aerogel-Based Adsorbents—A Critical Review” categorizes the aerogel compositions used for radionuclide removal [19,20] and the most suitable physicochemical characteristics for the scope. It concludes that the solution pH is the cardinal factor. Moreover, the functionalization of the aerogels is critical for an effective recovery in terms of selectivity.

“Improving the Performance of ZnS Photocatalyst in Degrading Organic Pollutants by Constructing Composites with Ag_2_O” studies the elevation of the photocatalytic activity of a nanocomposite derived from appropriate doping. The faster and more efficient degradation of methylene blue in comparison to the output of the single constituents is attributed to the combination of n-type and p-type semiconductors [21,22].

Another beneficial amalgamation is disclosed in “N-Doped Biochar as a New Metal-Free Activator of Peroxymonosulfate for Singlet Oxygen-Dominated Catalytic Degradation of Acid Orange 7”. The resulting recyclable nanocatalyst enhanced the peroxymonosulfate performance and dye decomposition rate by 38 times compared to the metal-containing counterparts [23]. The authors justified this based on a selective non-free radical, singlet oxygen activation mechanism [24].

An alternative implementation of magnetic nanomaterials, “Facile Synthesis of Magnetic Biochar Derived from Burley Tobacco Stems towards Enhanced Cr(VI) Removal: Performance and Mechanism”, reveals the impact of ferric oxide incorporation on burley tobacco stem biochar on the Cr(VI) adsorption capacity (14-fold increase). Moreover, the removal mechanism via the reduction of hexavalent chromium is further elucidated [25].

In the framework of reclaiming the raw materials abundant in nature, like nut shells [26,27], Ni-Doped Ordered Nanoporous Carbon Prepared from Chestnut Wood Tannins for the Removal and Photocatalytic Degradation of Methylene Blue describes an environmentally friendly method for preparing 2D hexagonally ordered carbon nanorods crosslinked by nickel cations. The latter efficiently adsorbed methylene blue, which also underwent photocatalytic degradation. They also exhibited good magnetic properties and recyclability.

Activated carbon, an already established potent adsorbent for toxic heavy metal ions [28] and dyes [29], was blended with an inorganic mixed oxide at the nanoscale via simple wet homogenization: Activated Carbon/ZnFe_2_O_4_ Nanocomposite Adsorbent for Efficient Removal of Crystal Violet Cationic Dye from Aqueous Solutions. The combination exhibited superior pollutant retention performance compared to its pure precursors, accomplished via a random physical and endothermic process.

The induction of the appropriate heterostructures in inorganic photocatalysts is a fruitful strategy for enhancing their activity [30,31]. A successful example is presented in Surfactant-Modified CdS/CdCO_3_ Composite Photocatalyst Morphology Enhances Visible-Light-Driven Cr(VI) Reduction Performance. Surface doping the cubic crystal structure of CdCO_3_ with CdS nanoparticles yields elliptical spheres exhibiting an increased specific surface area and excellent photocatalytic activity towards Cr(VI) reduction.

Inorganic composites in the form of nano-sized ceramic powders may promote photocatalytic degradation as well [32]. Evaluation of Photocatalytic Performance of Nano-Sized Sr_0.9_La_0.1_TiO_3_ and Sr_0.25_Ca_0.25_Na_0.25_Pr_0.25_TiO_3_ Ceramic Powders for Water Purification establishes this fact by examining pindolol decomposition under simulated solar irradiation via the mediation of free ^•^OH radicals [33]. 

Comparative Study of the U(VI) Adsorption by Hybrid Silica-Hyperbranched Poly(ethylene imine) Nanoparticles and Xerogels juxtaposes two different compositions of silica-hyperbranched poly-ethylene imine composites in the form of nanospheres [34] and xerogels [35]. The effects of the dendritic polymer quantity and substrate active surface are correlated with the retention of radioactive pollutants.

## 3. Conclusions

The development of specialized nanostructured materials for adsorption, separation, and purification has significantly enhanced the effectiveness of water depollution while reducing energy consumption and waste generation. This supports global goals for sustainability and environmental protection. Various kinds, including zero-valent metal nanoparticles (Ag, Fe, and Zn), metal oxides (TiO_2_, ZnO, and iron oxides), nanocarbons (such as carbon nanotubes, graphene oxide, and activated carbon), double-layered hydroxides, and nanomembranes, nanoceramics, and nanocomposites, are currently being used in wastewater treatment as adsorbents, (photo)catalysts, and antibacterial agents. Their distinct physicochemical characteristics offer significant benefits in the capture, extraction, and decomposition of numerous pollutants, including heavy metals, radionuclides, dyes, antibiotics, microbes, pesticides, and organic waste. Thus, nanotechnology is crucial for removing toxic substances from water. This collection compiles various case studies to inspire further research in this area.
